# Cognitive Modifiability of Mental Rotation: Effects of Mediation, Sex, and Working Memory Among Third-Grade Students

**DOI:** 10.3390/jintelligence14080162

**Published:** 2026-08-01

**Authors:** David Tzuriel, Ravit Hozez Sagi

**Affiliations:** 1 Faculty of Education, Bar-Ilan University, 2 Anna Webb Street, Ramat Gan 5290002, Israel; 2 Learning Disabilities Department, Talpiot Academic College, 7 Yotveta Street, Holon 5850019, Israel; 3 School of Education, Ariel University, 65 Ramat HaGolan Street, Ariel 4077625, Israel; hsravit@gmail.com

**Keywords:** mental rotation, working memory, cognitive modifiability, sex differences, mediated learning

## Abstract

The present study examined the effects of a brief motoric mediation intervention, delivered within a dynamic assessment (DA) framework, on mental rotation (MR) performance among third-grade children. The study also investigated the roles of sex and spatial working memory (SWM). Participants completed two MR tasks, a Windows Mental Rotation (WMR) test and the Seria-Think–MR (STI-MR) test, assessing generalization. Each task was administered before and after a single mediation session. Four major findings emerged. (a) Children who received individualized motoric mediation demonstrated significantly greater MR gains than control children on both tasks. (b) The intervention produced a generalization effect, with improvements observed even on the structurally distinct STI-MR task. (c) No significant sex differences or sex-related interactions were found. (d) The positive pre-intervention correlation between SWM and WMR weakened after mediation, suggesting reduced reliance on WM resources following the acquisition of more efficient MR strategies. Across both tasks, a consistent Degree of Rotation × Time interaction indicated disproportionately larger gains at higher rotation angles, and children showed evidence of strategic reframing, particularly in interpreting 270° rotations as equivalent to 90° in the opposite direction. These findings support the theoretical framework of Structural Cognitive Modifiability and highlight the potential of motoric mediation to foster flexible spatial strategies. The results also underscore the educational value of integrating spatially rich, hands-on activities into early curricula and demonstrate the diagnostic utility of DA for identifying children’s learning potential. Implications, limitations, and directions for future research are discussed.

## 1. Introduction

Mental rotation (MR) refers to the capacity to picture how an object would look after being turned in two- or three-dimensional space. Some researchers, such as Casey et al. (1995), view MR as an indicator of broader spatial reasoning skills. MR ability forms a core component of spatial reasoning and supports both academic learning and everyday problem-solving (e.g., Judd & Klingberg, 2021; Xu et al., 2025). Research spanning decades consistently shows substantial variation among individuals, including notable sex differences and a robust relation to spatial working memory (WM). Recent work further demonstrates that MR ability is highly trainable and that training can produce both behavioral gains and measurable neural changes. For example, Dong et al. (2025) showed that repeated mental rotation practice over 10 days led to significant and lasting improvements in accuracy and speed. Participants who trained consistently demonstrated stronger spatial-cognition performance even weeks after training ended. They also reported that pairing training with high-definition transcranial direct current stimulation (HD-tDCS) produced even more durable cognitive benefits, with neural measures (EEG and other electrophysiological markers) indicating strengthened spatial-processing networks after training.

Against this backdrop, the present study advances a dynamic, developmentally grounded approach to assessing and strengthening young children’s MR skills through mediated learning. By embedding both imagined and physical object transformations within a DA framework, the study captures not only children’s initial MR performance but also their modifiability—the degree to which they benefit from structured mediation designed to reveal and enhance latent learning potential. This approach allows us to address several open questions in the early spatial-cognition literature. Specifically, we examine whether sex differences in MR, widely reported in older populations, are already detectable in early childhood and whether such differences persist once mediation is provided. We also investigate whether boys and girls differ in their responsiveness to MR training, testing the possibility that structured mediation may reduce or eliminate initial performance gaps. Finally, we analyze how degrees of rotation shape both performance and modifiability, and how the relationship between spatial working memory and MR shifts from pre- to post-intervention. These issues are developed in the following sections, which review (a) sex differences in MR, (b) the interaction between training and sex, and (c) rotation angle effects and the evolving correlation patterns between WM and MR.

### 1.1. Sex Differences in Mental Rotation

Meta-analyses showed that most spatial-ability tests indicated sizable sex differences (e.g., Lauer et al., 2019; Linn & Petersen, 1985; Voyer et al., 1995). The strongest sex differences tend to emerge specifically when mental rotation (MR) is assessed with the Mental Rotation Test (MRT; Vandenberg & Kuse, 1978). Even so, MR is still described as the spatial skill with the most robust and well-documented male advantage (Hegarty, 2018). Research on MR consistently shows that sex differences are among the largest documented in spatial cognition, with males typically outperforming females across many MR tasks. This pattern is supported by both classic meta-analytic work and more recent empirical studies and meta-analytic interpretations. A foundational source is the classic meta-analysis by Voyer et al. (1995), which demonstrated that MR tasks produce robust, medium-to-large male advantages (effect sizes d = 0.56–0.73), larger than in other spatial abilities such as spatial perception or visualization. This foundational conclusion continues to be reaffirmed in modern research syntheses and empirical studies. More recent developmental evidence, summarized in Lauer et al. (2019), shows that sex differences emerge early and increase with age. In early childhood (around age 6), sex differences are small (g ≈ 0.20) but grow steadily through adolescence, reaching medium effect sizes (g ≈ 0.50) by age 14. This trajectory suggests that both biological maturation and social–cognitive influences contribute to the widening gap. Several recent studies further illuminate factors underlying these differences. Rahe et al. (2023) reiterate developmental meta-analytic conclusions showing a progression from small early differences to large adult differences. Important methodological insights come from Jost and Jansen (2024), who examined whether task design accounts for sex differences. Their results showed that features such as the number of alternatives and stimulus mirroring affected overall MR performance but did not replicate meaningful sex differences in their large sample. This suggests that specific task designs can minimize or eliminate observable sex gaps, highlighting the sensitivity of MR outcomes to test structure. In contrast, some recent studies continue to find persistent male advantages even under novel or immersive conditions. For example, Jacobs et al. (2024) reported that in fully immersive 3D virtual reality MR tasks, males showed a large accuracy advantage despite the absence of time constraints and the use of highly naturalistic 3D stimuli. This challenges earlier beliefs that naturalistic or 3D tasks would reduce sex differences. Wang et al. (2026) reported that improvement in MR following intervention was significantly greater at angles included in the training, compared to untrained angles. They also demonstrated that training eliminated sex differences. Before training, males showed a typical advantage over females, but after training, no significant sex differences remained. Finally, neural-level and strategy-based explanations are supported by Bersier et al. (2024), who found behavioral and fMRI evidence that men and women may rely on different cognitive strategies (i.e., object-based vs. effector-based). Women displayed greater activation in the somatosensory and inferior frontal regions during MR, suggesting reliance on body-based processing, which may result in slower or less-accurate performance in classic object-rotation tasks. This supports a cognitive-strategy account of sex differences rather than purely biological explanations.

### 1.2. The Effect of Degree of Rotation on MR Performance

MR performance is highly sensitive to angular disparity between stimuli, with extensive research demonstrating that larger rotation angles reliably produce slower response times and lower accuracy. Classic chronometric studies showed a near-linear increase in processing time as angular disparity increased from 0° to 180° (Shepard & Metzler, 1971; Cooper & Shepard, 1973), a pattern replicated across age groups and task formats (Jansen et al., 2013). This angle–difficulty relationship also plays a central role in MR interventions. Because higher rotation angles impose greater cognitive demands, they tend to show the largest gains following training. Several training studies report disproportionately greater improvements at large angular disparities, indicating that intervention enhances the efficiency of the mental transformation process itself rather than only improving task familiarity (Wright et al., 2008; Neubauer et al., 2010; Jansen & Kellner, 2015). In summary, these findings suggest that the rotation angle is both a diagnostic indicator of MR difficulty and a sensitive measure of training-related change, making it a theoretically meaningful parameter for evaluating MR development.

### 1.3. The Relation Between Mental Rotation and Working Memory

Multiple studies demonstrate that individuals with higher WM perform better on MR tasks (e.g., Hyun & Luck, 2007; Pardo-Vazquez & Fernández-Rey, 2012). Research by Zhao et al. (2026) reports that a higher WM capacity is associated with greater accuracy in mental rotation, especially as angular disparity increases. This finding aligns with earlier findings (e.g., Hyun & Luck, 2007; Kaufman, 2007; Pardo-Vazquez & Fernández-Rey, 2012) showing that WM supports the maintenance and manipulation of spatial information during rotation. Correlational research consistently shows that spatial working memory predicts mental rotation performance, even when controlling for other cognitive abilities. A replication study on the cognitive mechanisms of MR notes that correlational evidence supports the role of spatial WM in mental rotation, despite mixed findings from experimental manipulations (Ebert et al., 2024). Some research suggests that object WM may contribute to MR, whereas spatial WM shows stronger, more consistent correlations. A replication study (Ebert et al., 2024) highlights this tension but confirms that spatial working memory remains a reliable predictor of mental rotation ability. A 2025 review on MR emphasizes that visuospatial working memory is central to mentally transforming objects, as it temporarily stores and processes spatial information required for rotation.

### 1.4. Rationale of the Study

Assessing MR in early childhood is essential because it underpins the development of spatial reasoning and predicts later success in STEM learning, yet it remains largely overlooked in early years instruction. As Bruce and Hawes (2015) observed, MR is “a particularly undeveloped area of current mathematics curricula,” reflecting a persistent gap between research and practice. A broad body of recent work confirms that MR is both foundational and underassessed: it is consistently identified as a core spatial process that supports higher-order reasoning, mathematical achievement, and everyday problem-solving (Schenck & Nathan, 2024; Harris, 2023; Mix et al., 2016). Meta-analytic evidence further shows that spatial training, especially MR training, produces reliable and transferable gains in STEM-related outcomes, underscoring its educational importance (Uttal et al., 2013). Despite this, MR is rarely measured directly in school assessments, where it is often subsumed under broader spatial categories (Harris, 2023). Developmental research demonstrates that MR emerges early and can be reliably measured in infants and young children (Beckner et al., 2023), and that MR performance is intertwined with cognitive control processes (Wang et al., 2026), aligning with the argument that spatial skills form a flexible, malleable cognitive toolkit (Newcombe, 2018; Tzuriel & Egozi, 2010). The present study is situated within Vygotsky’s framework of mediated learning and the zone of proximal development, as well as the theory of Structural Cognitive Modifiability (Feuerstein et al., 1979; Tzuriel, 2021). Together, these perspectives emphasize that cognitive abilities are not fixed traits but can be reshaped through deliberate, mediated learning experiences, emphasizing the role of the mediator in helping learners internalize cognitive principles that generalize beyond the immediate task—precisely the kind of modifiability required for MR, a skill that involves coordinating spatial relations across changing perspectives. Aligned with this framework, the study adopts a DA approach, which examines not only children’s pre-mediation MR performance but also the extent to which they benefit from mediation. By embedding visuomotor training that integrates both imagined and physical object transformations, the DA procedure operationalizes SCM principles: it provides graduated, strategic support designed to reveal and enhance children’s latent learning potential. Given established links between motor coordination, spatial working memory (SWM), and executive functioning (Michel et al., 2011; Piek et al., 2007, 2012; Zhao et al., 2026), the study also examines how SWM contributes to MR performance. By evaluating the effects of training generalization, the effects of a short MR intervention, and exploring how rotation angle interacts with modifiability, this work offers a dual-purpose DA tool that both measures and strengthens children’s spatial reasoning, with potential implications for long-term educational practice.

### 1.5. Hypotheses

1. Children in the experimental group are expected to show greater gains in mental rotation (WMR) performance from pre- to post-teaching compared with children in the control group, reflecting a near-transfer effect of the intervention.
2. Children in the experimental group are expected to exhibit greater improvement from pre- to post-teaching on the STI-MR test than children in the control group, indicating a far-transfer effect of the intervention.
3. Consistent with prior research showing early sex differences in spatial processing and strategy use, boys are expected to outperform girls on both MR tasks (WMR and STI-MR) during the pre-intervention phase. However, because spatial skills are highly malleable and responsive to targeted instruction, the intervention is expected to reduce these initial disparities. Following the intervention phase, girls are expected to close the performance gap, leading to comparable MR outcomes between boys and girls.
4. MR performance in the full sample will be positively correlated across the pre- and post-intervention phases. However, the strength of this correlation is expected to decrease from pre- to post-intervention. This reduction is anticipated because, following the intervention, children are likely to develop more efficient strategies or greater familiarity with the MR tasks, thereby reducing WM’s relative contribution to MR performance.
5. Improvements in MR performance from pre- to post-intervention are expected to be greater at higher rotation angles. This prediction is grounded in theory and prior research suggesting that children benefit more from mediation when task difficulty increases, making larger rotation angles particularly sensitive to intervention effects.

## 2. Methods

### 2.1. Participants

Before the study began, approval was obtained from the Ethical Committee of the Ministry of Education, and parents provided consent for their children’s participation. The sample comprised 68 third-grade children (34 boys and 34 girls) drawn from four classes in four elementary regional schools in central Israel. These regional schools serve communities with diverse socioeconomic backgrounds, providing a heterogeneous pool of participants. To ensure internal validity and minimize selection bias, randomization was conducted within each class: boys and girls were randomly assigned to either the experimental or the control group, with equal numbers of boys and girls allocated to each condition. This stratified random assignment procedure ensured balanced gender representation across groups and controlled for potential classroom-level influences. The mean age in the experimental group was 8 years and 9 months (SD = 4 months), and in the control group 8 years and 10 months (SD = 4 months), with no significant difference between the groups, t(66) = 0.71, *p* = .48. Mothers’ years of education in the experimental and control groups were 14.44 (SD = 2.16) and 14.59 (SD = 1.84), respectively, t (66) = 0.30, *p* = .76. Fathers’ years of education in the experimental and control groups were 14.53 (SD = 1.88) and 14.35 (SD = 2.60), respectively, t (66) = 0.32, *p* = .75. Parents’ occupation level (low versus high) showed a similar distribution in the experimental and control groups for both mothers, χ_(1)_^2^ = 1.50, *p* = .22, and fathers, χ_(1)_^2^ = 1.47, *p* = .23. Parents’ occupation level (low versus high) by gender showed similar distribution of boys and girls, among mothers, χ_(1)_^2^ = 0.60, *p* = .81, and fathers, χ_(1)_^2^= 0.60, *p* = .81.

### 2.2. Measures

#### 2.2.1. The Windows Mental Rotation–Dynamic Assessment (WMR-DA)

**Figure 1:**
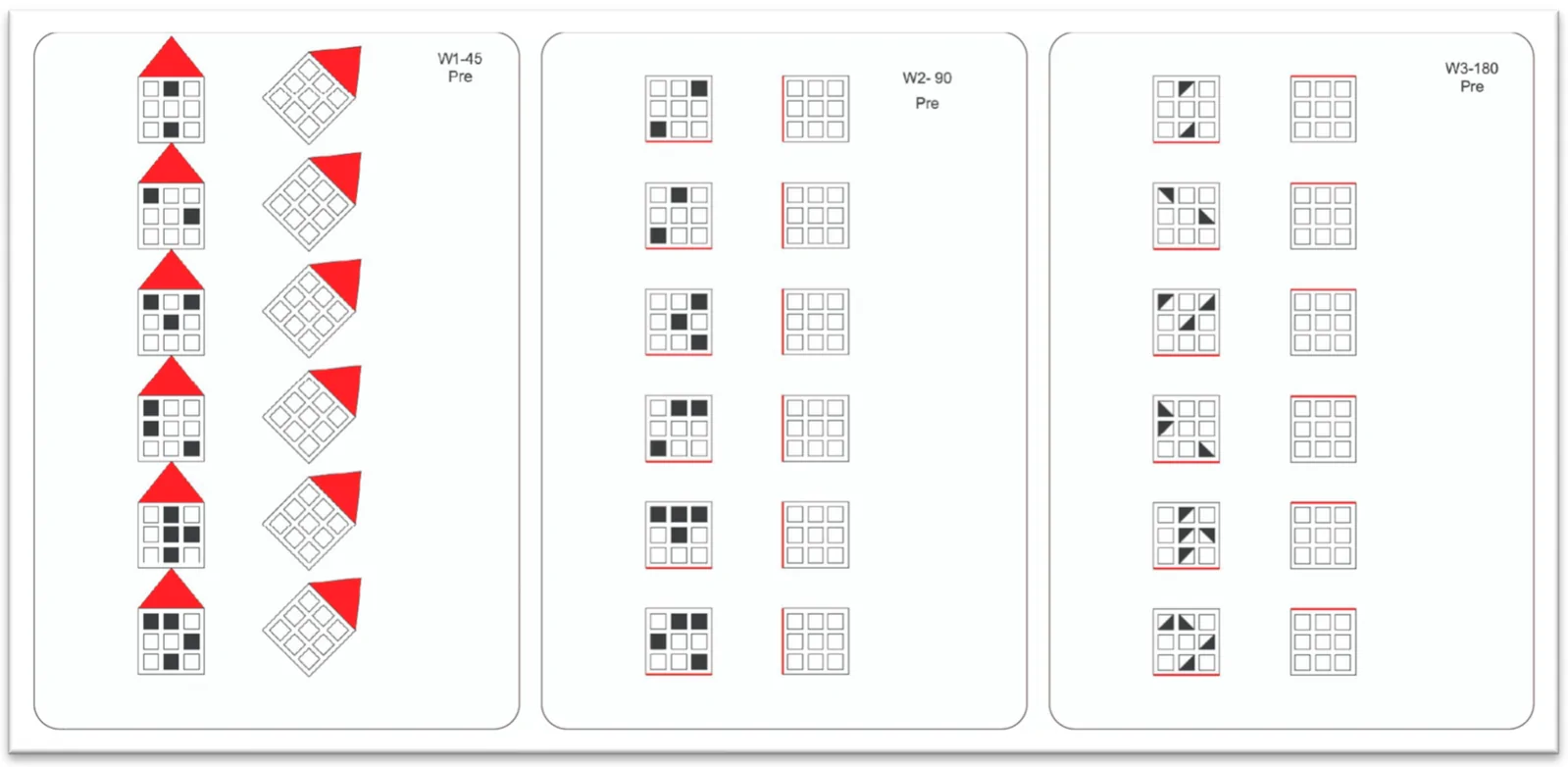
Examples of items from the Windows Mental Rotation–Dynamic Assessment (WMR-DA) Test (by the author’s permission).

The WMR-DA (Tzuriel & Egozi, 2010) was developed based on the mental rotation subtest of the Cognitive Modifiability Battery (Tzuriel, 1995, 2000; Tzuriel & Egozi, 2007). It is intended for children in first through fourth grades and includes three levels of difficulty (WT1, WT2, WT3). Each level has parallel forms administered during the pre- and post-intervention phases. Across all levels, children are shown model figures of “houses with windows” arranged in a 3 × 3 grid (nine windows), with some windows open and others closed (blackened). After the houses are rotated, children are asked to identify the corresponding closed windows on the rotated figure. Example items from Levels 1 (W1, 45°), 2 (W2, 90°), and 3 (W3, 180°) appear in Figure 1. In W1, the red roof provides a salient cue for determining the rotation, whereas in W2 and W3, the cue is a red line, which is less visually prominent. In W3 problems, children must not only identify the correct window after rotation but also mark the correct blackened half-square. Each level includes 18 items organized according to the degree of rotation required (45°, 90°, 180°), task complexity (two, three, or four closed windows), and symmetry (symmetrical or nonsymmetrical; see Figure 1). The unique structure of the WMR-DA enables separate analyses of performance by test level, rotation angle, complexity, and symmetry. Each correctly identified window receives a score of 1, yielding a maximum score of 54. In the present study, Cronbach’s alpha reliabilities for Levels 1–3 were 0.90 for the pre-intervention phase and 0.94 for the post-intervention phase. Reliability coefficients reported for a Grade 1 sample (Tzuriel & Egozi, 2010) were 0.78 and 0.73 for WT1, and 0.76 and 0.74 for WT2, for pre- and post-intervention, respectively. Construct validity was examined by analyzing performance as a function of task characteristics. Findings from a pilot study demonstrated a clear linear decrease in performance with increasing rotation angle, F(2, 111) = 503.09, *p* < .001, η_p_^2^ = 0.90; task complexity, F(2, 111) = 28.53, *p* < .001, η_p_^2^ = 0.34; symmetry, F(1, 112) = 342.83, *p* < .001, η_p_^2^ = 0.75; and test level (WT1 vs. WT2), F(1, 112) = 532.55, *p* < .001, η_p_^2^ = 0.83. Analyses of the initial composite WMR scores similarly revealed significant performance declines as a function of rotation angle, F(2, 111) = 416.80, *p* < .001, η_p_^2^ = 0.88, and test level, F(1, 112) = 366.70, *p* < .001, η_p_^2^ = 0.77 (Tzuriel & Egozi, 2010).

#### 2.2.2. The Seria-Think Instrument–Mental Rotation (STI-MR) Test

**Figure 2:**
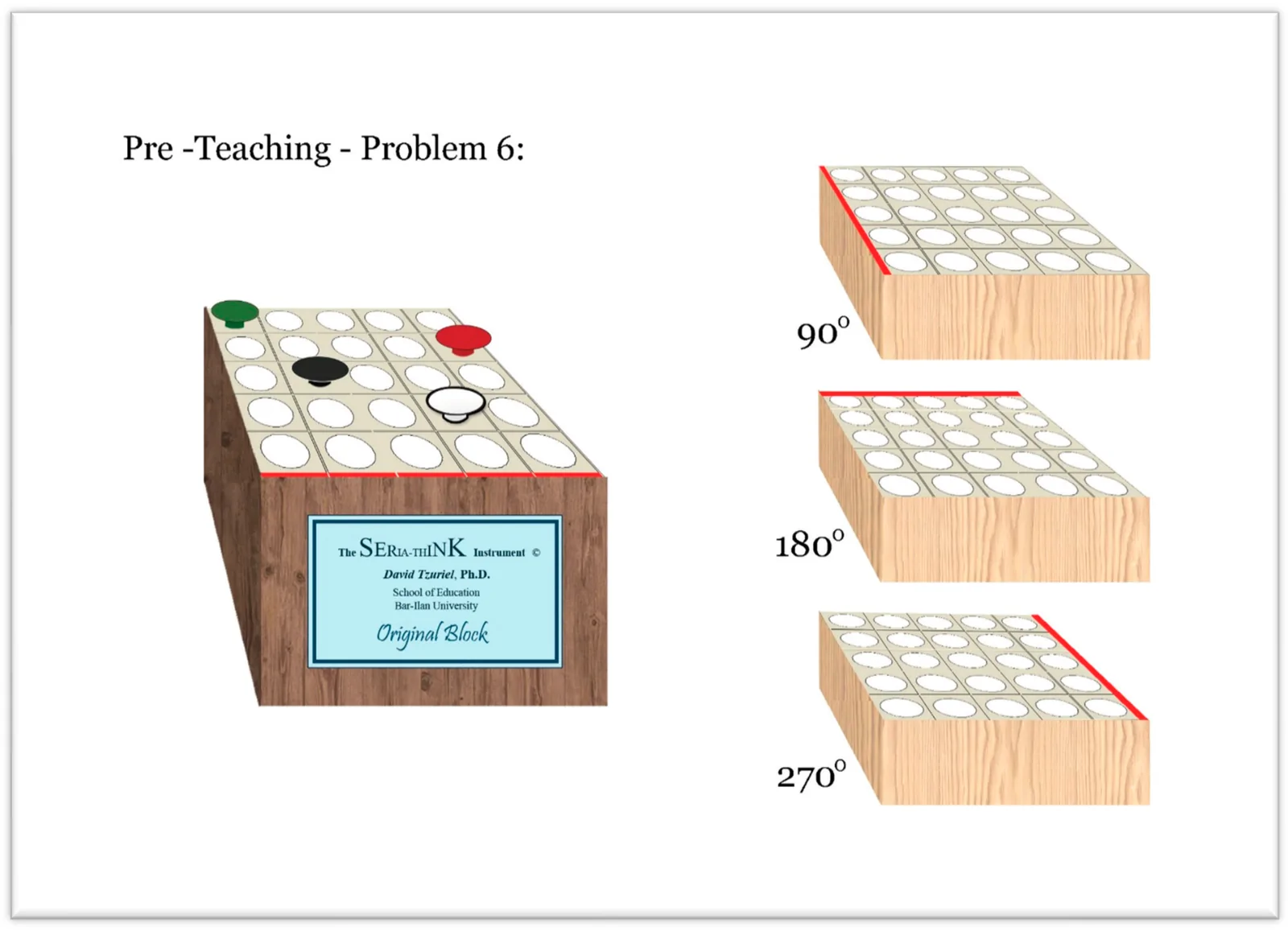
Example item from the Seria-Think Instrument–Mental Rotation test (STI-MR) (by the author’s permission).

The STI-MR test is a DA tool designed for use in clinical, educational, and research contexts. It assesses MR abilities in children aged 5 to 10 years. In the measurement/research format, the instrument includes three phases: pre-teaching, teaching, and post-teaching. During administration, the child is presented with the STI Original Block and the Transfer Block (see Figure 2), positioned side by side, with the Transfer Block to the right of the Original Block. The examiner places 2, 3, or 4 colored buttons into the holes of the Original Block and asks the child to place the same-colored buttons into the corresponding holes of the Transfer Block after the block has been rotated 90°, 180°, or 270°. An example item is provided before the pre-teaching phase begins. The child’s responses are recorded on the Recording and Scoring Sheet and later evaluated for accuracy. After each response, the examiner removes the buttons and rotates the Transfer Block for the next trial. Both the pre-teaching and post-teaching phases consist of 18 items. Each item varies along three dimensions: degree of rotation (90°, 180°, 270°), complexity (2, 3, or 4 buttons), and stimulus symmetry (symmetrical or asymmetrical). For each rotation angle, there are six items: two with two buttons (one symmetrical, one asymmetrical), two with three buttons (one symmetrical, one asymmetrical), and two with four buttons (one symmetrical, one asymmetrical). This structure allows for detailed analysis of children’s performance across each dimension, as well as total scores. The examiner instructs the child to place the buttons in the Transfer Block so that, after a single clockwise rotation (90°, 180°, or 270°), the button configuration matches that of the Original Block. In the present study, Cronbach’s alpha reliability coefficients were 0.88 for the pre-teaching phase and 0.87 for the post-teaching phase.

#### 2.2.3. The Children’s Spatial Working Memory (CSWM) Test

**Figure 3:**
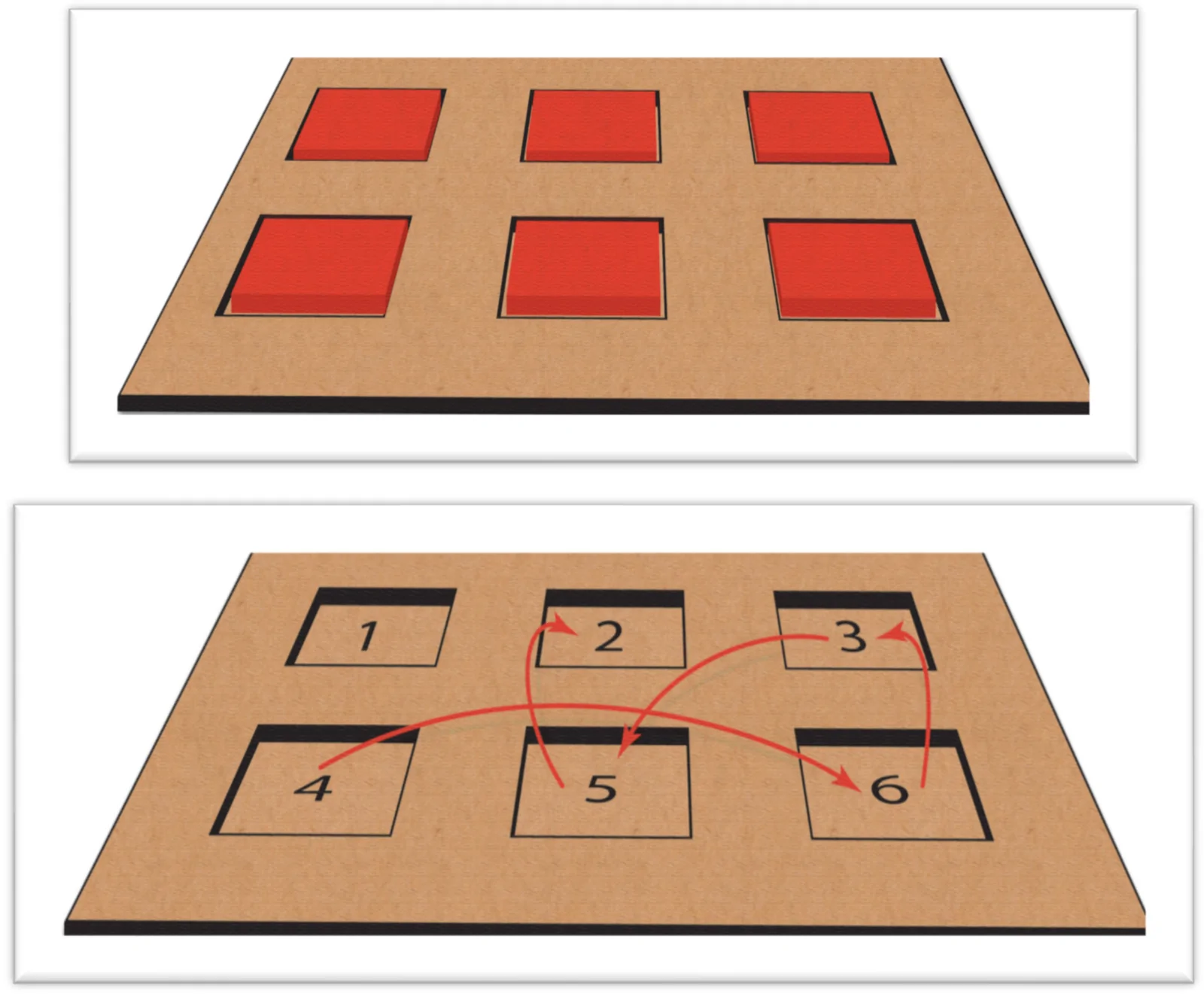
The Children’s Spatial Working Memory (CSWM) test (by the author’s permission). Note. The arrows in Figure 3 indicate the sequence in which the windows are presented to the child (4–6–3–5–2). The numbers are included for illustrative purposes only and do not appear on the test apparatus. Therefore, examiners were required to memorize the locations of the windows and their presentation order when administering the test items.

The CSWM test is a spatial WM measure designed for young children. It consists of a rectangular wooden board with two rows of three carved “windows” (3 × 2), each containing a red wooden square (see Figure 3). Spatial working memory is assessed by asking the child to recall and point to a predetermined sequence of spatial locations. The test includes seven levels, each comprising four trials. At each level, the examiner points to a series of windows—ranging from one to seven locations—thereby increasing task difficulty across trials. Testing is discontinued when the child fails three consecutive trials. Each correctly recalled sequence earns one point, and points are summed to produce a total score. The CSWM test was validated in studies using the Modifiability of Working Memory Program (MWMP; Tzuriel et al., 2024, 2026). It showed significant correlations with the Knox Cube Test (r = 0.48, *p* < .01; Richardson, 2005) and with the mental rotation subtest of the CMB (r = 0.55, *p* < .001; Tzuriel, 2000). Additional findings reported by Tzuriel et al. (2024) demonstrated significant correlations with the Children’s Verbal Working Memory Test (r = 0.34, *p* < .01; Tzuriel, 2019), the Backward Digit Recall test (r = 0.46, *p* < .01; Pickering & Gathercole, 2001), the Understanding of Directions subtest of the Woodcock–Johnson IV (r = 0.49, *p* < .01; Hoover & Davis, 2017), and the Head–Toes–Knees–Shoulders test (r = 0.44, *p* < .01; C. E. C. Ponitz et al., 2008; C. C. Ponitz et al., 2009).

#### 2.2.4. The Cognitive Modifiability Battery–Mental Rotation (CMB-MR) Subtest

**Figure 4:**
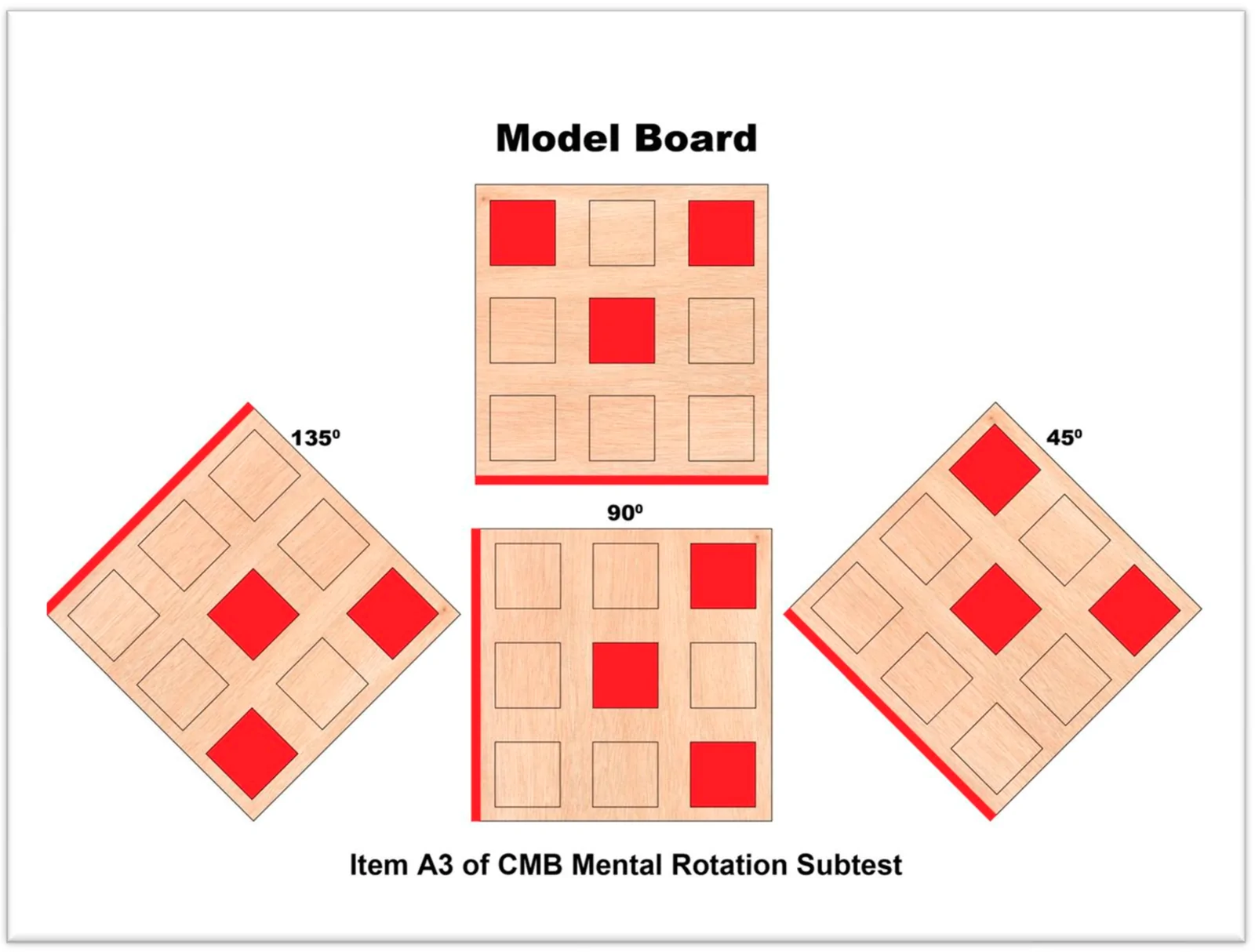
Example of an item from the CMB-MR subtest (by the author’s permission).

The CMB–MR subtest was used in the present study as the intervention instrument. An example item is shown in Figure 4. The materials include four CMB plates, each marked with a red strip along the base to help children track the progression of rotations from the model plate to the three rotated plates. Children are presented with six items: a model plate displaying a pattern of closed windows and three additional plates arranged horizontally from right to left, representing rotations of 45°, 90°, and 135°. Each item varies in the number of closed windows (2, 3, or 4) and in symmetry (symmetrical vs. nonsymmetrical). Figure 4 illustrates an item with all three rotation degrees, medium complexity, and a symmetrical pattern. To solve each item, the child must mentally rotate the model pattern according to the progressively rotated positions and motorically place wooden squares in the corresponding windows. During administration, the examiner places the plates in front of the child without the wooden squares. The child is instructed to reproduce the pattern of closed windows from the model plate onto each rotated plate. The teaching phase aims to develop strategies that help the child conserve the model pattern across rotated positions and motorically place the wooden squares into the windows. The child may take back the window piece and reposition it as many times as necessary until a satisfactory solution is reached. Six items are used during the intervention phase. Teaching strategies are adapted to the task demands and to the child’s response orientation (global vs. analytical), which is identified during the pre-teaching phase. For example, children may physically rotate the plates to verbalize the underlying principle in their own words (e.g., “when the plate moves, the windows inside the plate move with it; they do not move within it”) and to self-check their responses by rotating the plates back to the model’s original position. As children progress, instruction gradually shifts from physical rotation to mental rotation. Teaching emphasizes a holistic, global approach focused on reproducing the overall pattern. Children are encouraged to “visualize in the head” how the plate turns in space, using the red baseline as a visual anchor while reconstructing the pattern on the rotated plates. Clinically, this global strategy has proven effective for most children because it does not require labeling or analytically tracking directional changes. An additional verbal–analytic strategy may also be used, in which children label the positions of closed windows (e.g., “top-left,” “middle-bottom”). However, clinical experience indicates that the global approach is generally more efficient for solving mental rotation tasks. This is consistent with research showing that global-processing strategies are generally more efficient for solving mental rotation tasks than local, analytic approaches (Taragin et al., 2019; Tzuriel & Egozi, 2010).

### 2.3. Procedure

Before the start of the study, the College Ethical Committee and the National Ministry of Education Ethics Committee approved the study; parents provided written consent, and participation was voluntary. All children were tested by the CSWM, WMR, and the STI-MR tests in that order in a single session (75 min) before the intervention phase. The intervention phase, a single 45-min session, was administered 2 days after the pre-intervention phase and was provided only to the children in the experimental group. The intervention was administered by six graduate students enrolled in a Learning Disabilities program. Prior to working with the study participants, all mediators completed a structured training sequence consisting of four sessions (4 h in total), during which they were introduced to the principles of mediated learning and were specifically trained to administer the CMB-MR task. Consistent with recommendations for ensuring intervention fidelity in educational and cognitive-training research (Odom et al., 2010; Perepletchikova & Kazdin, 2005), the training included guided practice, modeling, and feedback. The visual–motor training with the CMB-MR test (Figure 4) was conducted using a global (holistic) processing approach. Children were first encouraged to perceive the overall configuration of the closed windows on the CMB-MR plate as a single visual pattern, and only then to use the red line cue relative to that pattern when determining the correct rotated position. Rather than focusing on individual windows separately, participants were trained to identify the spatial relations among all windows simultaneously and to mentally transform the entire configuration as a unified gestalt. This strategy was based on evidence from the global-processing literature indicating that perception and spatial problem-solving are often more efficient when guided by holistic pattern recognition than by a sequential analysis of isolated details. Research on global precedence demonstrates that observers typically process overall configurations before local elements, and that global representations facilitate visual recognition and spatial judgments (Kimchi, 1992; Navon, 1977; Gerlach & Poirel, 2018; Tzuriel & Egozi, 2010). Accordingly, training emphasized constructing an integrated mental representation of the pattern and using the red line as a stable orientation cue during mental rotation. Each graduate student also conducted two supervised practice sessions with children not in the study sample, allowing them to refine their mediation skills and ensure procedural consistency. This approach aligns with established guidelines in DA research, which emphasize the importance of mediator preparation and supervision to maintain reliability and standardization of the mediation process (Lidz & Peña, 2020; Tzuriel, 2021). The post-intervention phase, conducted on the same day, was identical to the pre-intervention phase. Children in the control group received the same set of problems to practice without teaching or explanations. The post-intervention phase was identical to the pre-intervention phase.

## 3. Results

### 3.1. Statistical Analysis Plan

The data were analyzed using repeated-measures ANOVA and Pearson correlations. Prior to statistical analyses, we conducted a sensitivity analysis using G*Power (3.1) (Faul et al., 2009). The analysis for the primary Treatment × Time interaction indicated that with *N* = 68 (34 per treatment group), α = 0.05, power = 0.80, and an assumed correlation among repeated measures of r = 0.50 (sphericity ε = 1.0), the minimum detectable effect size was Cohen’s f = 0.345 (no centrality parameter λ = 8.0837), corresponding to f^2^ = 0.119 and partial η^2^ = 0.106 (Richardson, 2011). This effect size is between medium and large by conventional benchmarks (approximately equivalent to Cohen’s d ≈ 0.69). We therefore had adequate power to detect effects of this magnitude.

### 3.2. WMR-DA Performance in Pre- and Post-Intervention: Effects of Treatment, Sex, and Degree of Rotation

**Figure 5:**
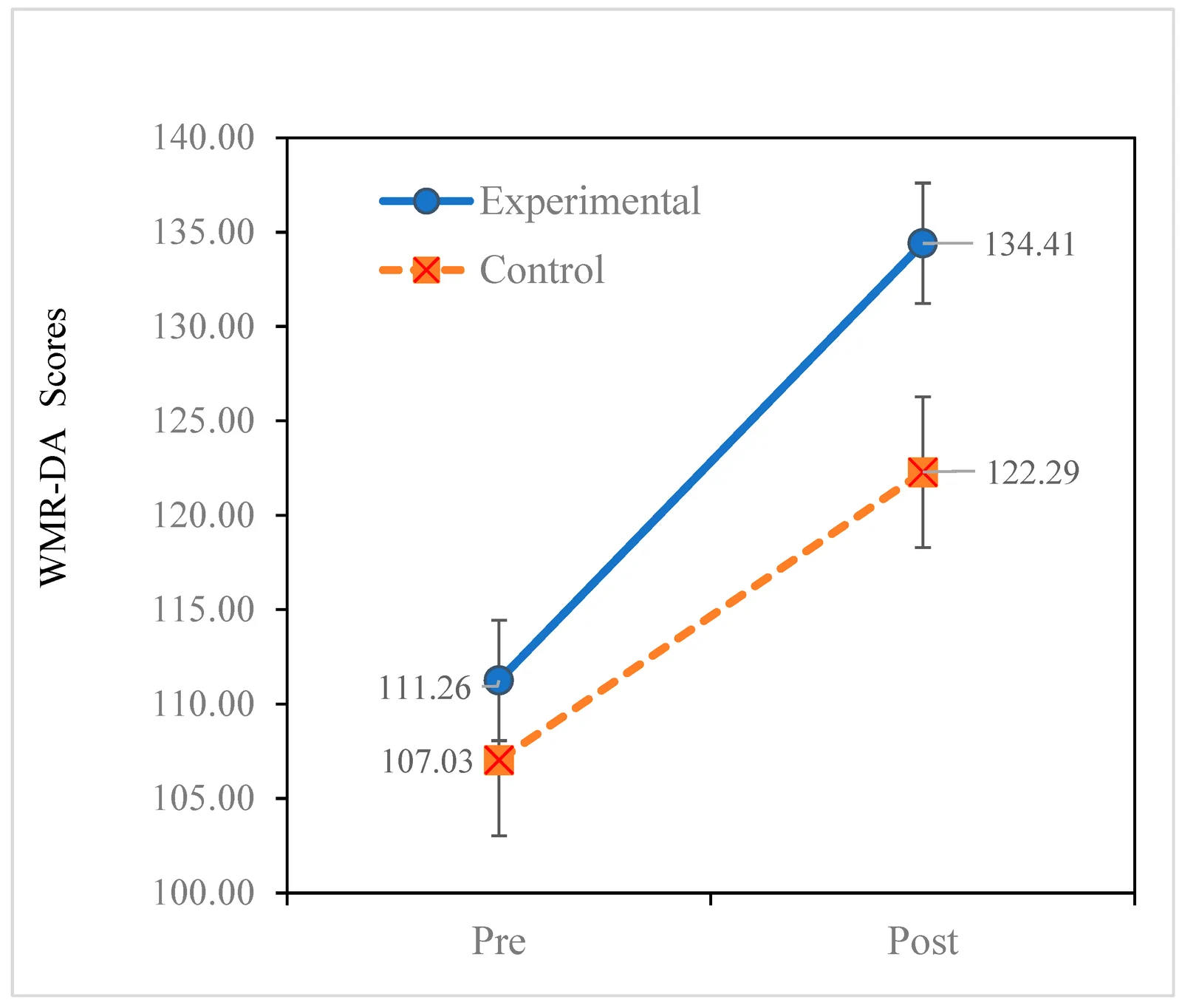
Pre- and post-intervention WMR-DA scores in the experimental and control groups.

**Figure 6:**
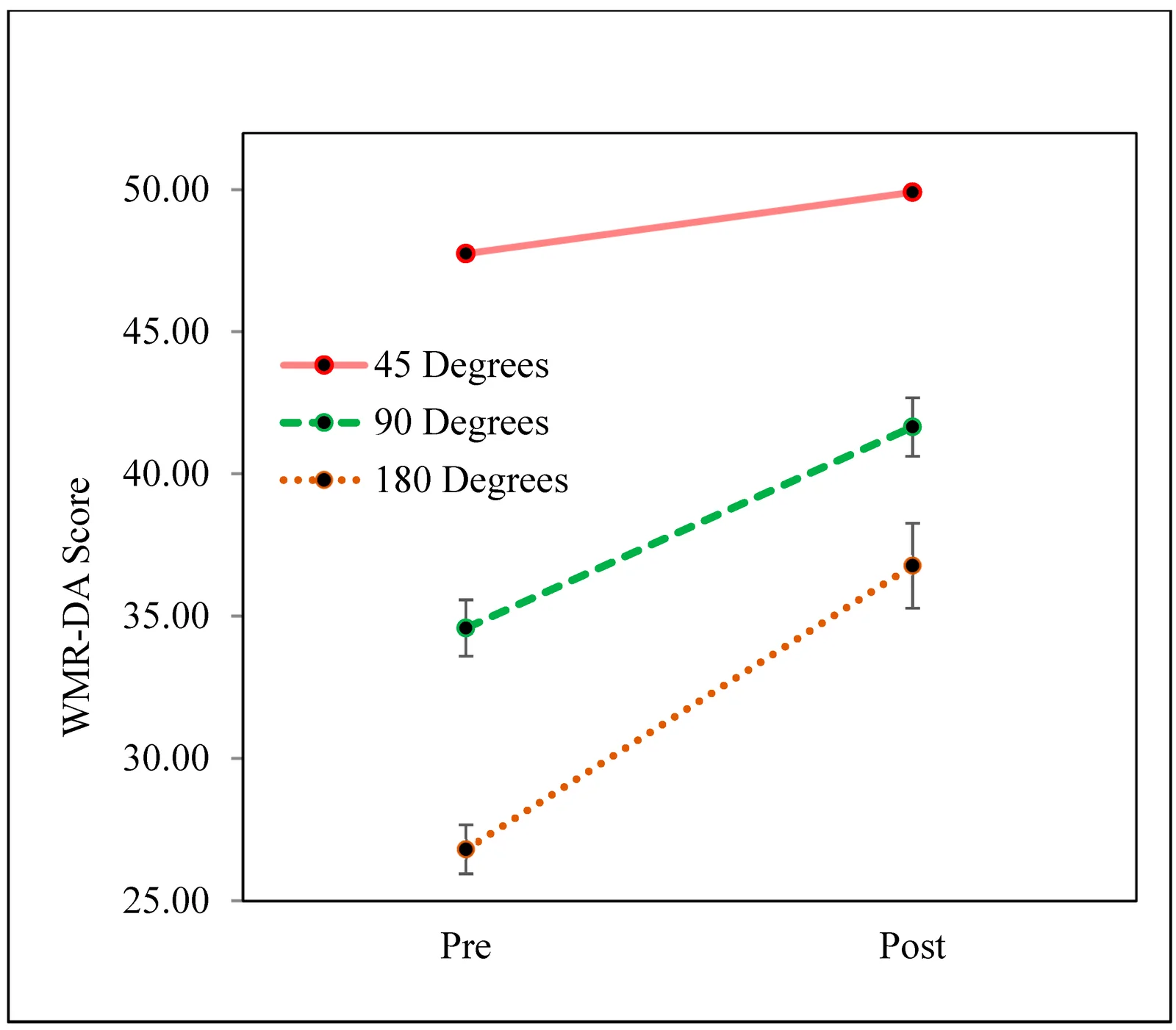
Pre- and post-intervention WMR-DA scores by degrees of rotation.

Table 1 presents the means and SDs of pre- and post-intervention WMR scores for the experimental and control groups, by sex. The data were analyzed using a repeated-measures nested ANOVA with a Treatment × Sex × Degree of Rotation × Time interaction (2 × 2 × 3 × 2), with time (pre/post) as the main effect nested within the degree of rotation. The findings (Table 2) show a significant main effect of degree of rotation and time, indicating, as expected, a linear decrease with higher rotation levels (45° M = 48.83, SD = 5.21; 90° M = 38.16, SD = 7.62; 135° M = 31.79, SD = 8.78), and higher scores in the post-intervention than in the pre-intervention phase. The pattern of results was qualified by significant Treatment × Time and Degree of Rotation × Time interactions (see Figure 5 and Figure 6, respectively).

Follow-up tests on the Treatment × Time interaction indicated that both groups improved from pre- to post-intervention: the experimental group showed a large and highly significant gain, *t*(33) = 6.33, Cohen’s dz = 1.09, *p* < .001, and the control group also improved significantly, *t*(33) = 4.17, Cohen’s dz = 0.72, *p* < .001. Between-group comparisons confirmed that the groups did not differ at baseline, *t*(66) = 0.94, Cohen’s d = 0.23, *p* = .35, but diverged at post-intervention, with the experimental group outperforming the control group, *t*(66) = 2.14, Cohen’s d = 0.52, *p* =.036 (post-test between-group Cohen’s d ≈ 0.52, medium effect). In sum, although both conditions showed pre-to-post gains, the intervention produced a larger improvement, yielding a significant group advantage.

Post hoc analyses with Bonferroni adjustment were conducted to clarify the significant interaction between the degree of rotation and time in the repeated-measures nested design (η_p_^2^ = 0.78). Within each rotation condition, participants demonstrated significant improvements from pre- to post-intervention. Performance increased from 47.75 (*SE* = 0.73) to 49.91 (*SE* = 0.62) at 45°, *p* < .001, Cohen’s d = 0.39; from 34.59 (*SE* = 0.99) to 41.66 (*SE* = 1.03) at 90°, *p* < .001, Cohen’s d = 0.85; and from 26.81 (*SE* = 0.86) to 36.78 (*SE* = 1.49) at 180°, *p* < .001, Cohen’s d = 0.99. Thus, the intervention produced progressively larger effects as the rotation angle increased. Comparisons among rotation angles indicated that at both pre- and post-intervention, performance differed significantly across all rotation angles after Bonferroni correction, with the highest scores at 45°, followed by 90° and then 180° (all *p* < .001). Importantly, although overall performance decreased as the rotation angle increased, the magnitude of improvement was larger at higher rotation angles, with the greatest gains observed at 180°. These results indicate that the intervention produced robust improvements across all rotation conditions, with disproportionately larger benefits at more challenging rotation angles. The robustness of these effects is supported by the strong correlation between pre- and post-intervention WMR scores (r = 0.75, *p* < .001), indicating substantial rank-order stability and reduced within-subject error variance, thereby strengthening confidence in the reliability of the observed post hoc effects.

### 3.3. STI-MR Performance in Pre- and Post-Intervention: Effects of Treatment, Sex, and Degree of Rotation

**Figure 7:**
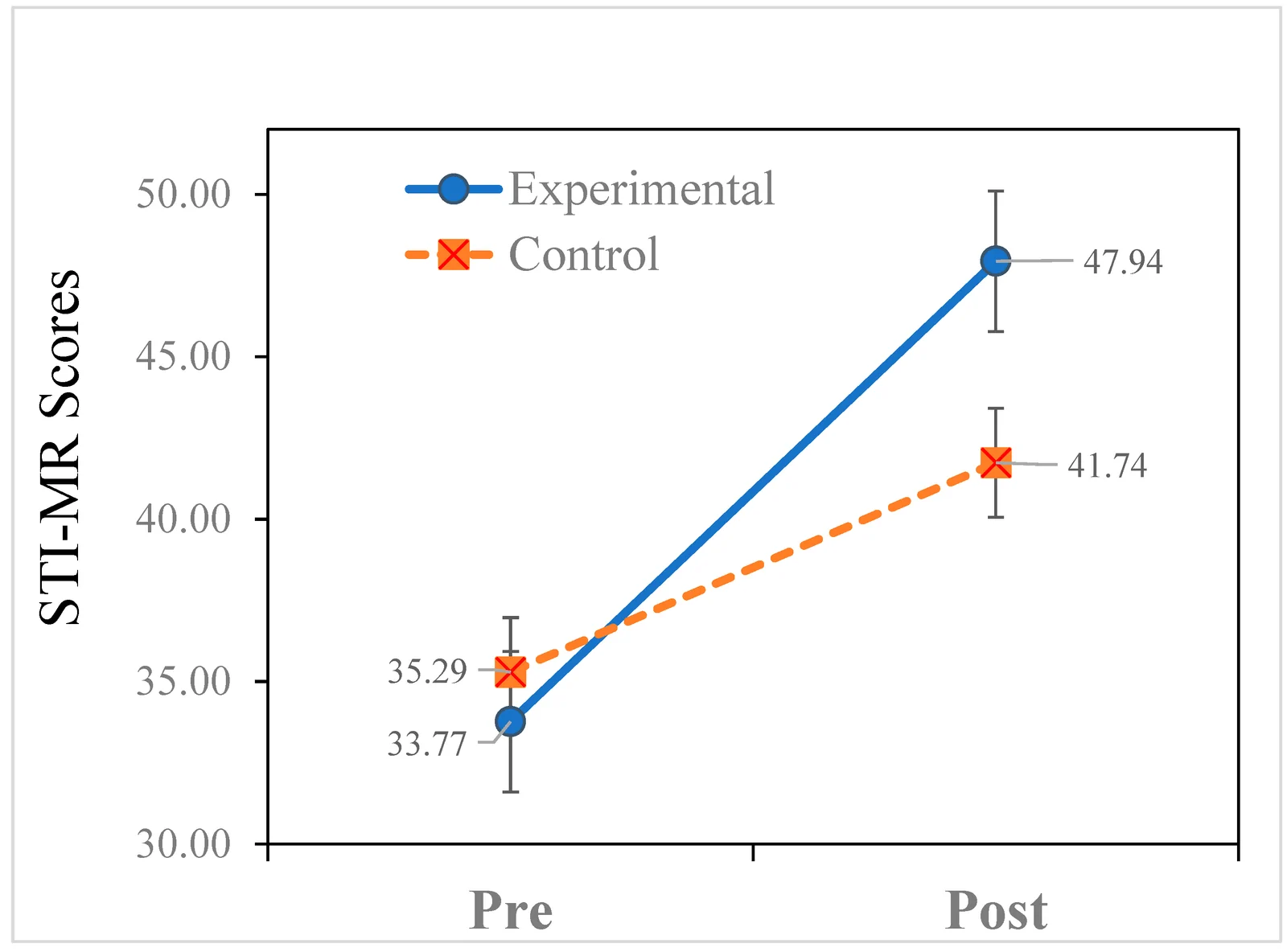
Pre- and post-intervention STI-MR scores in the experimental and control groups.

**Figure 8:**
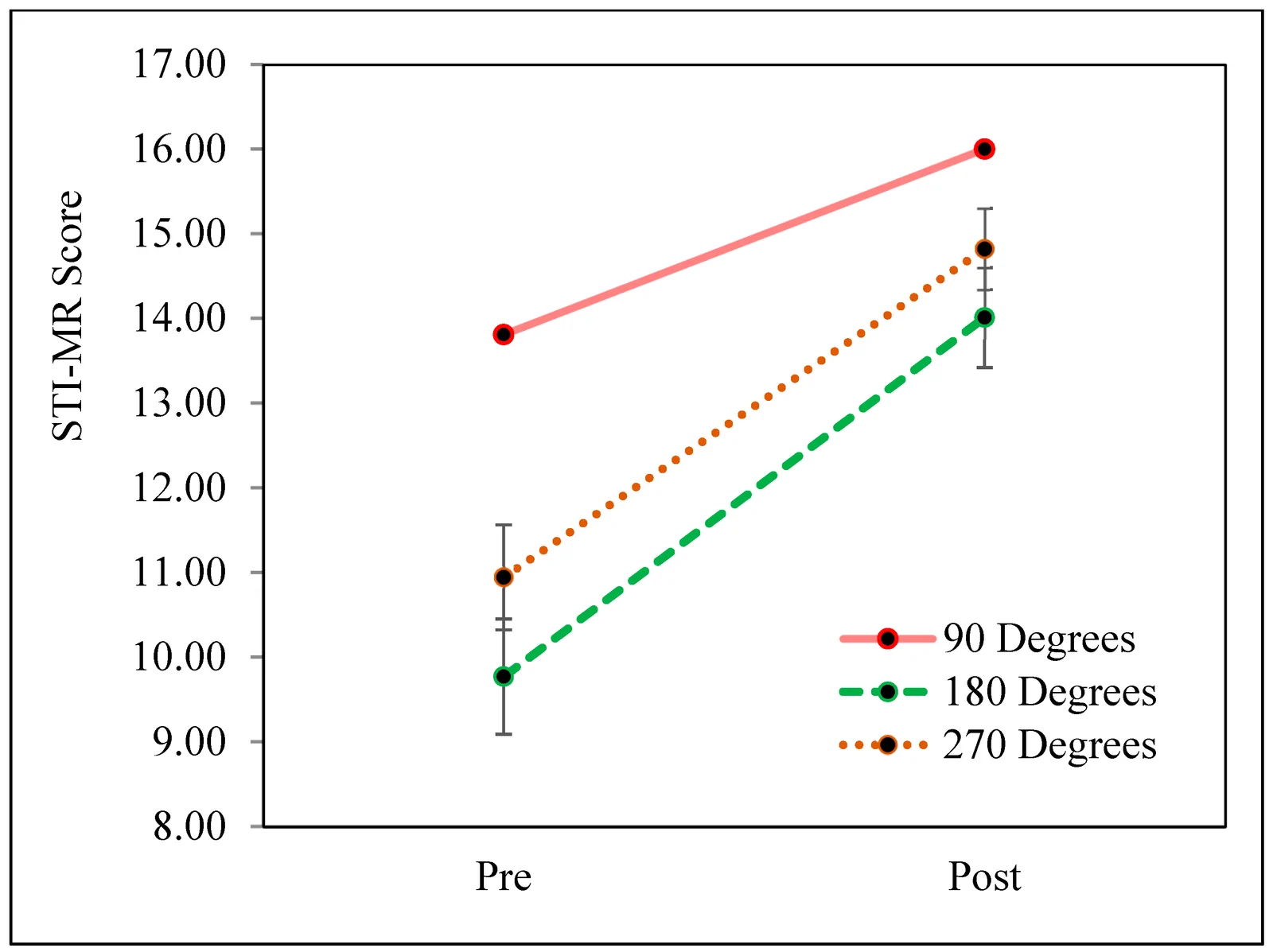
Pre- and post-intervention STI-MR scores by degree of rotation.

STI-MR performance is considered a measure of generalization for the MR intervention. The data were analyzed using a repeated-measures nested ANOVA with a Treatment × Sex × Degree of Rotation × Time interaction (2 × 2 × 3 × 2), with time as the main effect nested within the degree of rotation. The findings (Table 2) reveal, as expected, a significant main effect of the degree of rotation. This finding indicates an interesting pattern: there was a decrease from 90° (M = 14.90, SD = 2.97) to 180° (M = 11.90, SD = 5.12); however, the score at 270° (M = 11.95, SD = 4.48) was about the same as at 180°. The main effect of time, as expected, indicates higher scores in the post-intervention phase than in the pre-intervention phase. These findings were qualified by significant Treatment × Time and Degree of Rotation × Time interactions (see Figure 7 and Figure 8, respectively).

Post hoc comparisons were conducted to follow-up on the significant Treatment × Time interaction. Between-group t tests indicated no baseline difference between groups, t(66) = −0.47, *p* = .64, Cohen’s *d* = −0.11. At the post-intervention phase, the experimental group outperformed the control group, t(66) = 2.61, *p* = .011, Cohen’s *d* = 0.63. Paired t tests (assuming a pre–post correlation of *r* = 0.50) showed significant gains in both groups: experimental t(33) = 7.21, *p* < .001, Cohen’s *dz* = 1.24; control t(33) = 3.33, *p* = .002, Cohen’s *dz* = 0.57. Thus, although both treatment groups improved, the experimental group showed a larger improvement, yielding a significant group advantage at the post-intervention phase. As in the analyses of the WMR, the robustness of these post hoc analyses is supported by the strong correlation between pre- and post-intervention STI-MR scores (r = 0.68, *p* < .001), indicating substantial rank-order stability and reduced within-subject error variance, thereby strengthening confidence in the reliability of the observed post hoc effects.

Post hoc analyses with Bonferroni adjustment were conducted to clarify the significant interaction between the degree of rotation and time in the repeated-measures nested design. Within each rotation condition, participants demonstrated significant improvements from pre- to post-intervention. Performance increased from 13.81 (*SE* = 0.40) to 16.00 (*SE* = 0.37) at 90°, *p* < .001, Cohen’s *d* = 0.69; from 9.77 (*SE* = 0.68) to 14.01 (*SE* = 0.59) at 180°, *p* < .001, Cohen’s *d* = 0.81; and from 10.94 (*SE* = 0.62) to 14.82 (*SE* = 0.48) at 270°, *p* < .001, Cohen’s *d* = 0.65. Comparisons among rotation angles indicated that at both pre- and post-intervention, performance differed significantly across all rotation levels after Bonferroni correction, with the highest scores at 90°, followed by 270°, and then 180° (all *p* < .001). Importantly, although overall performance decreased as the rotation angle increased from 90° to 180°, the magnitude of improvement was greater at higher rotation angles, with the greatest gains observed at 180° and 270°. These findings indicate that the intervention produced robust improvements across all rotation conditions, with disproportionately larger benefits at more demanding rotation angles.

### 3.4. The Relationship Between Working Memory and Mental Rotation

A particularly compelling theoretical insight emerges from the marked shift in the relationship between spatial working memory (SWM), as assessed by the CSWM test, and mental rotation (MR) from pre- to post-intervention. The findings are presented in Table 3.

The findings clearly show significant correlations between SWM and MR tests in the pre-intervention phase; however, the decrease in correlation was found only for the WMR-DA scores. A sensitivity analysis indicated that, with a sample of 68 children, the study could reliably detect correlations of approximately *r* ≥ 0.33 with 80% power, meaning that smaller post-intervention correlations (e.g., *r* = 0.14–0.21) fell below the detectable threshold. Likewise, the minimum detectable difference between pre- and post-intervention correlations was approximately Δ*r* ≈ 0.28–0.30, indicating that only relatively large shifts in association could be identified with confidence. Thus, the non-significant change in the STI-MR correlation should be interpreted cautiously, as the study was underpowered to detect small-to-moderate differences.

## 4. Discussion

The present study investigated the effects of motoric mediation within a DA framework on MR performance among third-grade students, as well as the effects of sex and spatial working memory (SWM). Three major findings emerged: (1) children who received individualized motoric mediation showed significantly greater MR gains than control children on both the WMR and STI-MR tests, indicating generalization effects beyond the intervention task; (2) no significant sex differences or sex-related interactions were observed; and (3) the positive pre-intervention correlation between MR and SWM weakened after intervention, suggesting reduced reliance on working memory resources following the acquisition of efficient MR strategies.

### 4.1. Effectiveness of Motoric Mediation

The significant Treatment × Time interaction on both MR measures confirms that a brief, individualized motoric mediation session can meaningfully enhance MR ability in young children. This finding is consistent with the broader DA literature, which holds that cognitive abilities are not fixed traits but are modifiable through mediated learning experiences (Feuerstein et al., 1979; Tzuriel, 2001, 2013, 2021). The mediation protocol used in this study, which involves the physical manipulation of plates, verbal articulation of rotation principles, and a gradual transition from physical to mental rotation, reflects core principles of Structural Cognitive Modifiability (Tzuriel, 2013, 2021). Recent work on embodied cognition further supports this approach (Tzuriel et al., 2024). Research demonstrates that physical engagement with learning materials helps bridge the gap between concrete action and abstract reasoning, particularly in young learners (Gizzonio et al., 2021). The present findings align with Dong et al. (2025), who showed that repeated MR practice produced lasting behavioral and neural gains, though the current study uniquely demonstrates that even a single session of mediated motoric intervention can yield measurable improvement.

Importantly, the control group was not a passive no-treatment comparison group. Rather, children in this condition were given the same materials and opportunities for task engagement as the experimental group but received no strategic mediation, explanations, feedback, or guided instruction. Consequently, improvement in the control group can reasonably be attributed to practice, familiarity with the task format, repeated exposure, and motivational engagement. The fact that the experimental group nevertheless demonstrated significantly larger gains on both MR measures is therefore theoretically important, as it suggests that mediation provided benefits beyond those attributable to attention, engagement, or repeated practice alone. At the same time, because both groups improved significantly, the findings indicate that a portion of the observed gains reflects general practice effects, and the intervention effects should be interpreted as incremental gains above and beyond those practice-related improvements. This finding is not surprising given that mere exposure to MR tasks can produce practice effects (Uttal et al., 2013).

### 4.2. The Effect of Mediation on Generalization

A particularly noteworthy finding is that the intervention produced a generalization effect on both the WMR and the STI-MR tasks. The STI-MR test differs from the WMR in several ways: it uses a three-dimensional physical apparatus, involves different rotation angles (90°, 180°, and 270° rather than 45°, 90°, and 180°), and requires the child to physically place buttons rather than mark windows on paper. The emergence of generalization effects on this structurally different task is theoretically significant, as it suggests that the mediation fostered genuine cognitive modifiability (i.e., the internalization of transferable MR strategies) rather than task-specific learning. One possible explanation for why the current study succeeded in producing a generalization effect is that the mediation focused not merely on practicing MR but on building conceptual understanding of the spatial transformation process, a deeper level of learning that is more likely to generalize across contexts (Uttal et al., 2013; Gilligan et al., 2017). The motoric component, focusing on physically rotating plates and observing the consequences, may have provided a multi-sensory representational foundation that supported transfer, consistent with embodied cognition perspectives (Newcombe, 2018). The findings reflect short-term modifiability rather than the durability of learning. DA is intended to reveal learners’ responsiveness to mediation, but determining whether these gains persist, generalize, or translate into broader educational outcomes requires a much more intensive intervention followed by delayed post-tests and longitudinal follow-up. Recent research on cognitive and spatial-training interventions underscores this point. Studies on mental rotation training show that although short-term improvements are common, long-term maintenance is not guaranteed and must be empirically verified through follow-up assessments. For example, Dong et al. (2025) found that mental rotation training effects diminished over time, even though some benefits were still detectable up to 90 days post-training, highlighting the need for extended monitoring to understand the trajectory of learning gains. These findings align with the broader cognitive-training literature, which emphasizes that short-term gains do not necessarily translate into long-term educational impact without sustained intervention and longitudinal evaluation (e.g., Sala & Gobet, 2019).

### 4.3. Rotation Angle Effects

The significant main effect of rotation angle across both measures replicated the well-established finding that MR difficulty increases with angular disparity (Shepard & Metzler, 1971; Cooper & Shepard, 1973; Jansen et al., 2013). More importantly, the significant Degree of Rotation × Time interactions suggest that improvements from pre- to post-intervention increased with rotation angle, with larger gains observed at higher angles. This pattern is consistent with prior training research showing that larger improvements tend to emerge at more demanding rotation angles (Wright et al., 2008; Neubauer et al., 2010). Importantly, this suggests that mediation enhanced the efficiency of the underlying mental transformation process itself, rather than merely fostering superficial familiarity with the task format. Across both the WMR and STI-MR tasks, a consistent pattern emerged: the degree of rotation interacted with time, indicating that the intervention produced improvements across all rotation conditions, with larger gains at more demanding rotation angles. This parallel interaction across tasks suggests that the cognitive mechanisms underlying mental rotation are similarly responsive to training, regardless of the tasks’ structural similarity. Notably, in the STI-MR task, performance at 270° exceeded performance at 180°, a pattern that initially appears counterintuitive given the greater angular disparity. However, this finding aligns with children’s strategic reinterpretation of the 270° rotation: many recognize that a 270° turn is functionally equivalent to a 90° rotation in the opposite direction, allowing them to rely on a simpler and more familiar transformation. This strategic reframing reduces cognitive load and supports more efficient processing, thereby explaining the higher performance at 270° relative to 180°. Together, these results indicate that children not only benefit from the intervention but also adaptively refine their rotation strategies, leading to enhanced performance across both near- and far-transfer contexts.

The finding that the most difficult rotation showed the largest gains provides converging evidence for the cognitive modifiability interpretation: it is precisely when the task demands are highest that the availability of internalized strategies makes the greatest difference.

The convergence of findings across the WMR and STI-R tasks carries important theoretical implications for understanding how children acquire and generalize MR skills. Recent work emphasizes that MR development reflects both improvements in spatial working memory and the refinement of flexible transformation strategies rather than a single unitary ability (Frick & Pichelmann, 2023; Mix et al., 2022). The parallel rotation-by-time interactions observed here suggest that the intervention strengthened a shared underlying mechanism—likely the ability to coordinate spatial relations across increasing angular disparities—while also promoting adaptive strategy selection. The disproportionately large gains at higher rotation angles in both tasks indicate that children became more efficient at handling cognitively demanding transformations, consistent with accounts proposing that practice accelerates the shift from effortful analytic processing to more holistic or rule-based strategies (Uttal & Cohen, 2012). The specific advantage at 270° in the STI-R task further illustrates this strategic flexibility: children appear to spontaneously recode a 270° rotation as a simpler 90° transformation in the opposite direction, demonstrating an emerging metacognitive awareness of rotational equivalence. This ability to restructure spatial problems aligns with contemporary models of spatial transfer, which argue that far-transfer gains arise when learners internalize generalizable principles rather than task-specific procedures (Zhu et al., 2023). Thus, the present findings add to a growing body of evidence demonstrating that targeted spatial training enhances the efficiency and modifiability of children’s mental rotation (MR) processes, thereby supporting broader spatial reasoning beyond the trained tasks.

### 4.4. Absence of Sex Differences

Contrary to much of the existing literature, no significant main effects or interactions involving sex were found in the current study. This null finding contrasts with meta-analytic evidence documenting reliable male advantages in MR, particularly with the Vandenberg and Kuse MRT (Voyer et al., 1995; Lauer et al., 2019). Several factors may account for this result. Here are some possible reasons for the absence of sex differences, either initially or as a result of training.

First, there is emerging evidence that sex differences in MR have narrowed in some recent cohorts, and several sociocultural mechanisms have been proposed to explain this trend. However, the pattern is not uniform, and the evidence is still developing. Recent findings suggest that gender differences in MR may be less stable across contemporary cohorts than previously assumed. Although classic work typically reports a male advantage, several recent studies indicate that sociocultural factors, such as stereotype activation, spatial self-concept, and increased spatial experiences for girls, can substantially modulate or reduce these differences. For example, stereotype-related processes have been shown to shape performance in adolescents, with girls’ spatial self-concept mediating the impact of stereotype activation on MR outcomes (Rahe et al., 2023). Training-based interventions can also eliminate gender gaps, demonstrating the malleability of these differences under supportive conditions (Tzuriel & Egozi, 2010; Wang et al., 2026). At the same time, other recent studies continue to document robust male advantages under specific task conditions, including variations in task complexity among primary-school children (Reuter & Reinhold, 2025) and in 3D VR environments (Jacobs et al., 2024). Overall, the emerging pattern suggests that while sociocultural influences and increased awareness of gender stereotypes may be narrowing the gap in some populations, the presence and magnitude of sex differences remain strongly dependent on task design, cognitive demands, and contextual factors. Second, the participants were third-grade children (mean age approximately 8:9), an age at which sex differences in MR are still relatively small (g ≈ 0.20; Lauer et al., 2019), compared with the larger differences observed in adolescence and adulthood. Third, as Jost and Jansen (2024) have demonstrated, specific task features, such as stimulus type, number of alternatives, and mirroring, can substantially affect whether sex differences emerge. The WMR and STI-MR tasks use simplified, child-appropriate stimuli (house-window patterns and button placements, respectively) rather than the complex three-dimensional block figures associated with the largest sex differences. Consistent with the present results, Ebert et al. (2024) found no sex differences in a replication study of WM and MR using simpler stimuli. Fourth, Wang et al. (2026) demonstrated that MR training can eliminate previously observed sex differences, suggesting that initial male advantages may reflect differential experience rather than fixed cognitive capacity. From a DA perspective, the absence of sex differences is consistent with the view that cognitive modifiability, when appropriately mediated, can transcend individual-difference variables that appear substantial in static assessments (Tzuriel & Egozi, 2010).

### 4.5. How the Relationship Between Working Memory and Mental Rotation Changes with Training

One of the most theoretically intriguing findings concerns the shift in the association between spatial working memory (SWM), as measured by the CSWM, and MR from pre- to post-intervention. Prior to the intervention, SWM showed significant positive correlations with MR performance on both the WMR (*r* = 0.32) and the STI-MR (*r* = 0.27) tests, consistent with well-established links between SWM and MR ability (Hyun & Luck, 2007; Kaufman, 2007; Pardo-Vazquez & Fernández-Rey, 2012). After the intervention, however, these correlations weakened (WMR: *r* = 0.14, *ns*; STI-MR: *r* = 0.21, *ns*), with the Fisher Z test confirming a significant reduction for the WMR. This pattern is consistent with theories of skill acquisition, suggesting that as individuals develop efficient task-specific strategies, they rely less on general-purpose cognitive resources such as WM (Velázquez-Vargas & Taylor, 2024). In classic Shepard-type MR tasks, performance improvements are thought to reflect a shift from computationally demanding algorithmic processing (which taxes WM) to more efficient, possibly retrieval-based or automatized strategies (Logan, 1988). The mediation provided in the present study, particularly the emphasis on seeing patterns holistically and using visual cues, may have facilitated precisely this kind of strategy shift. Ebert et al. (2024) highlighted the enduring tension between experimental and correlational evidence regarding the respective roles of spatial versus object WM in MR. It is interesting to note that the reduction in correlation from pre- to post-intervention was significant only for the WMR task. This difference might clarify the distinction between the two generalization MR tasks—WMR and STI-MR. Although the WMR task is presented in a 2D format, it is visually aligned with the intervention task in terms of stimulus structure (3 × 3 grid), number of complexity levels (three), and number of symmetry types (two). This similarity likely enabled children to apply the MR strategies acquired during the intervention phase without heavily relying on WM. In contrast, the STI-MR task differs substantially from the intervention tool, particularly in its stimulus configuration (a 5 × 5 grid) and complexity pattern (the number of buttons dispersed across a 5 × 5 array). These visual and structural differences may have limited children’s ability to transfer the learned MR strategies, thereby preventing a reduction in WM demands from pre- to post-intervention. The present findings add a developmental–interventional dimension to this debate, suggesting that the association between SWM and MR is not static but dynamic, diminishing as children internalize more efficient spatial strategies through mediated learning. It is important to note that the secondary analyses comparing pre- and post-intervention correlations had less statistical power than the primary intervention effects. A sensitivity analysis indicated that with a sample of 68 children, the study had adequate power to detect correlations of approximately r ≥ 0.33, and differences between dependent correlations of roughly Δr ≈ 0.28–0.30. Consequently, smaller post-intervention associations, such as those observed for the STI-MR task, fell below the detectable threshold, and non-significant changes should therefore be interpreted with caution. This limitation underscores the need for larger sample sizes in future work to more precisely evaluate shifts in the relationship between SWM and MR following mediation.

### 4.6. Theoretical and Educational Implications

The findings carry several theoretical and practical implications. Theoretically, they support the concept of Structural Cognitive Modifiability (Feuerstein et al., 1979; Tzuriel, 2013) by demonstrating that MR—a foundational spatial ability—is substantially modifiable through brief, targeted mediation, even in young children. The evidence of generalization is particularly significant, as it suggests that mediated learning produces structural changes in cognitive functioning rather than surface-level improvements. The present findings offer clear practical implications for educational settings, particularly for designing interventions that strengthen children’s spatial reasoning. The consistent rotation-by-time interaction across both the WMR and STI-R tasks indicates that children benefit most when instruction provides opportunities to practice mental transformations across a wide range of angular disparities, including more challenging rotations. The disproportionately large gains at higher rotation angles suggest that, when appropriately scaffolded, difficult transformations can accelerate the development of flexible spatial strategies. Educators can support this growth by incorporating activities that prompt children to compare rotation paths, recognize rotational equivalences (such as understanding that 270° is functionally a 90° turn in the opposite direction), and verbalize or externalize their strategies. These practices align with current recommendations for spatially rich instruction, which emphasize the explicit use of strategies, guided discovery, and hands-on manipulation of objects in multiple orientations. Practically, the results underscore the value of integrating spatially oriented, motoric mediation activities into early childhood curricula, where mental rotation remains underrepresented despite its established role in supporting STEM learning (Bruce & Hawes, 2015; Judd & Klingberg, 2021; Schenck & Nathan, 2024; Xu et al., 2025). The present study demonstrates that even a single, well-designed session can yield meaningful and transferable gains, making such interventions feasible within typical school constraints. Moreover, the DA approach used here provides diagnostic insight by revealing children’s learning potential beyond what static tests capture (Tzuriel, 1995, 2021), offering educators a powerful tool for identifying and supporting students who stand to benefit most from spatial training.

### 4.7. Cognitive Developmental Implications

Beyond demonstrating the trainability of mental rotation, the present findings have implications for contemporary theories of cognitive development and intelligence. Current developmental perspectives increasingly emphasize that cognitive growth reflects changes in the efficiency, flexibility, and coordination of cognitive processes, rather than the unfolding of fixed abilities alone (Frick & Pichelmann, 2023; Zhu et al., 2023). From this perspective, mental rotation can be viewed as a developing cognitive tool whose effectiveness depends not only on spatial representations but also on the strategies children use to transform and manipulate those representations.

The finding that the experimental group showed greater gains than an active-practice control group is particularly important in this regard. Because both groups received equivalent exposure to the tasks, the additional gains observed following mediation are unlikely to be explained solely by familiarity or repeated testing. Instead, they suggest that children benefited from acquiring more efficient strategies for representing and transforming spatial relations. Such findings are consistent with developmental accounts proposing that cognitive improvement often reflects qualitative changes in strategy use and representational organization rather than simple increases in processing capacity.

Particularly noteworthy is the reduction in the association between spatial working memory and WMR performance following mediation. Before the intervention, children with stronger spatial working memory tended to perform better on mental rotation tasks. After mediation, however, this relationship weakened substantially. One interpretation is that mediation enabled children to rely less on effortful maintenance and manipulation processes, and more on internalized spatial strategies. This pattern is consistent with theories of cognitive development that suggest learning promotes a transition from resource-demanding processing to more efficient and coordinated forms of cognition.

These findings also resonate with Feuerstein’s Structural Cognitive Modifiability theory and Vygotskian’s perspectives on development. Both frameworks propose that cognitive development occurs through guided participation in culturally organized learning experiences that help children acquire new modes of thinking. The present results suggest that mediation may accelerate the development of spatial reasoning by helping children internalize principles of spatial transformation that can then be applied across tasks. In this sense, intervention does not merely improve performance on a specific spatial task; it may also facilitate changes in the cognitive mechanisms underlying intelligent behavior.

More broadly, the findings support the view that aspects of intelligence are developmentally malleable and responsive to structured educational experiences. Mental rotation requires the flexible coordination of spatial representation, executive control, and strategic processing, all of which are central components of higher-order reasoning. Therefore, interventions that strengthen these underlying processes may contribute to broader cognitive development and provide a foundation for later learning in mathematics, science, engineering, and other STEM domains.

### 4.8. Limitations of the Study and Future Directions

Several limitations should be acknowledged. First, the sample comprised 68 typically developing children from a single region in Israel, limiting the generalizability of the findings. Replication with larger, more diverse samples is needed, including children from varied socioeconomic and cultural backgrounds, as well as children with learning difficulties, who may benefit differently from mediation. Second, the study examined only immediate post-intervention effects. A more intensive intervention with longitudinal follow-up is required to determine the durability of the gains and whether they transfer to mathematics or other academic domains; the absence of a delayed post-test constrains conclusions about the persistence of intervention effects. Third, mediation was delivered individually, consistent with DA methodology, but this format may limit scalability in broader educational settings. Future studies should investigate whether group-based or technology-assisted adaptations of the mediation protocol can produce comparable results. Fourth, the CSWM test was administered only at pre-intervention, so it was not possible to assess whether the intervention also affected WM performance. Including WM measures at both time points would permit a more detailed examination of how the relationship between MR and WM evolves through mediation. Finally, while the current study focused on rotation angle as a task parameter, future research should examine how complexity and symmetry dimensions interact with mediation effects across different age groups and ability levels.

### 4.9. Conclusions

The present study shows that a brief, individualized motoric mediation session within a DA framework can meaningfully enhance children’s MR performance and support generalization across tasks. Consistent rotation-by-time interactions in both the WMR and STI-MR tasks suggest that mediation strengthened a core spatial transformation mechanism while promoting more flexible strategy use, particularly at higher angular disparities. Children demonstrated not only improved accuracy but also strategic reframing—such as reducing a 270° rotation to a simpler 90° transformation—indicating adaptive shifts in spatial reasoning. The reduced association between SWM and WMR-DA following mediation further implies that children adopted more efficient, less resource-dependent strategies. The absence of sex differences aligns with the view that cognitive modifiability, when appropriately mediated, can transcend individual-difference factors typically observed in static assessments. These conclusions should be interpreted with caution, given the modest sample and the focus on immediate post-intervention outcomes. Even so, the findings provide evidence that a single, well-structured mediation session can yield generalized improvements in spatial transformation skills. Future research should examine the durability of these gains, their potential transfer to academic domains, and the feasibility of more scalable formats, such as group-based or technology-supported mediation, across diverse populations and ability levels.

## Figures and Tables

**Table 1:** Means and standard deviations of the Windows Mental Rotation–Dynamic Assessment (WMR-DA) test by treatment (experimental/control), sex, and time (pre/post-intervention).

			WMR		STI-MR	
Phase	Treatment	Sex	M	SD	M	SD
Pre-Intervention	Experimental	Boys	112.88	17.13	34.94	11.91
		Girls	109.65	15.44	32.59	11.40
		Total	111.26	16.14	33.76	11.54
	Control	Boys	107.29	19.55	36.65	13.93
		Girls	106.76	22.43	33.94	13.57
		Total	107.03	20.72	35.29	13.61
Post-Intervention	Experimental	Boys	134.88	19.09	46.53	5.79
		Girls	133.94	15.10	49.35	5.52
		Total	134.41	16.96	47.94	5.75
	Control	Boys	122.00	27.67	43.65	13.12
		Girls	122.59	29.73	39.82	12.14
		Total	122.29	28.28	41.73	12.60

**Table 2:** Repeated-measures ANOVA of the Windows Mental Rotation (WMR) test by treatment, sex, time, and degree of rotation.

		WMR				STI-MR			
Source of Variation	df	MS	F	*p*	η_p_^2^	MS	F	*p*	η_p_^2^
Treatment (A)	1	757.67	2.88	.10	0.04	61.96	0.84	.364	0.01
Sex (B)	1	12.10	0.05	.83	0.00	26.00	0.35	.556	0.00
A × B	1	12.71	0.05	.83	0.00	34.71	0.47	.496	0.01
Error	64	790.21							
Degree of Rotation (C)	2	10,085.47	248.40	.001	0.80	319.68	36.40	.001	0.36
A × C	2	99.83	2.46	.090	0.04	2.46	0.51	.603	0.01
B × C	2	1.03	0.03	.975	0.00	1.33	0.15	.860	0.00
A × B × C	2	31.57	0.56	.518	0.01	0.16	0.02	.981	0.00
Error	128	40.60				8.78			
Time (D)	1	4180.48	100.66	.001	0.61	1204.41	100.13	.001	0.61
A × D	1	176.04	4.24	.044	0.06	169.53	14.09	.001	0.18
B × D	1	8.25	0.20	.657	0.00	11.70	0.97	.328	0.02
A × B × D	1	0.98	0.02	.878	0.00	28.06	2.33	.132	0.04
Error	64	41.53				12.03			
C × D	2	529.81	39.57	.001	0.39	40.59	10.14	.001	0.14
A × C × D	2	27.72	2.07	.130	0.03	5.19	1.30	.277	0.02
B × C × D	2	16.55	1.24	.294	0.02	3.18	0.80	.45	0.01
A × B × C × D	2	8.11	0.61	.547	0.01	6.94	1.74	.18	0.03
Error (C × D)	128	13.39				4.00			

**Table 3:** Pearson correlations between Children’s Spatial Working Memory (CSWM) and mental rotation scores in pre- and post-intervention.

Test	Pre-Intervention	Post-Intervention	Fisher *Z*
WMR-DA	*r* = 0.32, *p* < .01	*r* = 0.14, *p* = 0.30	3.20, *p* < .002
STI-MR	*r* = 0.27, *p* = .02	*r* = 0.21, *p* = .08	−1.10, *p* > .050

## Data Availability

The original data presented in the study are openly available in the Research Dataset and Participants Characteristics at https://docs.google.com/spreadsheets/d/1tGSm5PFJ4BiRNT4JFipnnLYi1N7TTxOJ/edit?usp=sharing&ouid=111133703187341072847&rtpof=true&sd=true (accessed on 14 July 2026).
